# HIV-associated multicentric castelman disease, a report of 5 cases

**DOI:** 10.1186/1742-4690-9-S1-P136

**Published:** 2012-05-25

**Authors:** Sylvie Jonckheere, Jean-Cyr Yombi, Anne Vincent, Leila Belkhir, Dunja Wilmes, Bernard Vandercam

**Affiliations:** 1 Medecine Interne Infectiologie at Centre Refrence St Luc Ucl, Bruxelles, Belgium

## Introduction

Multicentric Castleman’s disease (MCD) is a rare, non-clonal lymphoproliferative disorder characterized by constitutional symptoms, anaemia and generalised lymphadenopathy.

## Aim

The present study intends to compare demographic features, clinical presentation, laboratory studies, imaging results as well as treatment regimens and out- come in our MCD patients to those of larger reported series.

## Method

We reviewed the files of 930 HIV-1-infected patients from our AIDS Reference Centre. Data was collected from the operating software for the patients’ medical records of our institution.

## Results

We report a series of five cases of MCD among our HIV/AIDS patients’ cohort. Three were of African origin. They were diagnosed after 2003, after a mean duration of 85 months of HIV-seropositivity. All presented with characteristic clinical features and laboratory findings (table 1), and all but one patient were started on HAART only a few months before or upon MCD diagnosis. Four patients were treated with chemotherapy, and one with HAART only. One patient who was given Adriamycin/Bleomycin/Vinblastin is in continuous remission after 6 years of follow-up. Two are alive, with good symptom control, regardless of the treatment they received. One recently relapsed, and one unfortunately died before completing the intended 6-courses chemotherapy regimen.

## Conclusion

MCD is a rare, but rising issue among HIV-infected patients. The clinical and paraclinical features of our series of five patients are in keeping with those of larger reported series. Currently, treatment is mainly chemotherapy-based, but a wide variety of protocols have been used, mainly because of the lack of available evidence. New approaches such as anti-CD 20 antibodies seem highly effective, and the role of HHV-8 needs to be further investigated, as it might be an important target for future treatment.

